# How does China’s green credit policy affect the innovation of high-polluting enterprises? From the perspective of innovation quantity and quality

**DOI:** 10.1371/journal.pone.0302789

**Published:** 2024-05-20

**Authors:** E. Bai, Kai Wu, Hongxin Zhu, Hejie Zhu, Zhijiang Lu

**Affiliations:** 1 School of Management, Harbin University of Commerce, Harbin, Heilongjiang, China; 2 School of Finance, Southwest University of Finance and Economics, Chengdu, Sichuan, China; Lincoln University, NEW ZEALAND

## Abstract

Employing the “Green Credit Guidelines” implemented in 2012 as the basis for a quasi-natural experiment, this study applies the method of Difference-in-Differences(DID) to investigate the influence of the Green Credit Policy on both the quantity and quality of enterprise innovation. The outcomes of our analysis reveal that the policy has significantly boosted both the quantity and quality of innovation among enterprises identified as heavy polluters. It is noteworthy that the policy’s positive impact on innovation quantity surpasses its positive effect on innovation quality. This substantiates that the Green Credit Policy effectively generates incentivizing outcomes for innovation among the heavy polluters, thereby verifying Porter’s hypothesis within the domain of green credit in China. Furthermore, we find that the positive impact is more significant for enterprises with lower innovation capabilities, large-scale enterprises, state-owned enterprises, and those situated in both the Eastern and Western regions. Through these findings, this study illuminates a novel perspective on the interplay between the Green Credit Policy and enterprise innovation dynamics in China.

## 1. Introduction

With increasingly prominent global environmental issues, the global spotlight has shifted to the realms of the green economy [1]. Being counted among the global foremost generators of carbon emissions, China assumes a critical role in tackling environmental challenges and promoting green transformation. The 19th National Congress of the Communist Party of China (CPC) clearly stated, "adhering to the harmonious coexistence between humanity and nature and building an ecological civilization is a millennium-long plan for the sustainable development of the Chinese nation." Furthermore, the 20th National Congress of the CPC once again elevated "harmonious coexistence between humanity and nature" to one of the connotations of "Chinese-style modernization," indicating China’s profound commitment to environmental protection. However, China currently primarily relies on adjusting its economic structure and accelerating technological progress to reduce carbon emissions, and the economic and technological measures adopted have not effectively achieved the anticipated carbon reduction targets [2]. Therefore, to bring about fundamental environmental change, reliance on financial tools is imperative. Financial instruments can guide, incentivize, and regulate the economic behavior of microeconomic entities, thereby facilitating a better realization of economic sustainability and environmental protection. Specifically, through fund allocation by commercial banks, financial instruments can direct social capital into green and environmentally friendly industries while restricting funds from flowing into highly polluting sectors. This approach not only facilitates the promotion of economic development but also assists highly polluting enterprises in transitioning from traditional business models, accelerating technological innovation, and achieving transformation and upgrading. Hence, the development of green financial instruments is not only a necessary means to improve the quality of China’s economic development but also represents the future direction of financial institutions.

Although the "Guidance on Building a Green Financial System" explicitly outlines that green finance encompasses financial products like green loans, green bonds, green insurance, and green development funds, it is challenging in this study to comprehensively estimate the role of green finance in all the above areas due to limitations in data availability. Simultaneously, according to estimates by CICC Research, the ratio of green credit balance to the overall green financing balance surpasses 90% [2]. Taking green bonds as an example, as of 2021, the scale of China’s green finance market exceeded CNY 17 trillion, with a green credit balance of CNY 15.9 trillion, ranking first globally, and the green bond balance of CNY 1.1 trillion, ranking second globally. While the scale of China’s green bond balance ranks second globally, it is less than one-tenth of the green credit balance [3]. Other forms of green financial financing represent even less than one percent of the total green finance amount [4]. Therefore, this study contends that research conclusions regarding green credit can to a large extent represent the role of green finance, as green credit constitutes the largest component within the realm of green finance.

The green credit policy revolves around the provision of preferential loans and financial support by financial institutions to enterprises that meet environmentally friendly standards while imposing credit constraints or raising credit thresholds for enterprises that fail to meet environmental requirements. It can channel resources toward the realm of environmental preservation, promote enterprise transformation, and reduce environmental pollution and resource waste. The eventual objective of the policy is to revolutionize the backward business models in China, where economic progress has historically resulted in environmental deterioration, with the aim of breaking the detrimental cycle of "pollute first, treat later" and "pollute again, treat again". The policy primarily influences enterprise technological innovation through the following three channels: financing, signaling, and reducing innovation risks. Firstly, in terms of financing, financial institutions remain the primary external financing channel for enterprises in China currently. They play a crucial role in providing loan funds to Chinese enterprises. Green credit, by controlling external financing channels for enterprises, increases their financing rates, compresses their financing space, and shortens their loan terms, ultimately raising the financing costs for heavy-polluting enterprises in China. On the other hand, from the perspective of enterprises, heavy-polluting enterprises in China, given their relatively large scale and high debt-to-asset ratios compared to other enterprises, rely more heavily on financial institutions, making financial institutions the most important financing channel for them [5]. Therefore, heavy-polluting enterprises in China are significantly affected by financial institutions’ credit policies. Following the implementation of green credit policy, enterprises’ "environmental performance" and "green performance" become crucial criteria for financial institutions to assess their loan quotas and terms. If heavy-polluting enterprises in China continue their original production methods without engaging in technological innovation and reforms, they will face increased difficulty in obtaining credit from financial institutions in the short term, leading to higher financing thresholds and ultimately exacerbating their difficulties in obtaining loans in the long term. Therefore, to ensure long-term development, these enterprises must engage in technological innovation and operational upgrades to reduce pollution levels and meet the loan requirements of green credit [6]. Secondly, from the perspective of signaling, since the Fifth Plenary Session of the 18th Central Committee of the Communist Party of China (CPC) from 26 to 29 October 2015, the Chinese government has been advocating the concept of green development, and since the 18th CPC National Congress, the concept of green development has been elevated to the height of national strategy. As another concrete practice of the green development concept, the green credit policy once again sends a signal to the whole society that "all people participate in the green economy". This prompts various sectors of society to pay more attention to the "environmental performance" and "green performance" of heavy-polluting enterprises. Under the supervision of the public, government, and other public opinions, China’s heavily polluting enterprises, to maintain their corporate image and achieve long-term development, are forced to adopt more environmentally friendly and efficient production methods through the renovation and upgrading of existing production processes and equipment, as well as the innovation of production technology. Eventually, through technological transformation upgrading, and innovation, heavily polluting enterprises can reduce pollutant emissions and improve resource utilization efficiency, which not only maintains the good image of the enterprise but also achieves sustainable development. Lastly, in terms of reducing enterprise innovation risks, innovation often accompanies certain risks, requiring enterprises to invest substantial amounts of capital, with uncertain outcomes [7]. Enterprises face high risks during innovation primarily due to the uncertainty of investment funds and the unpredictability of innovation results. Innovation projects may require significant capital investment, but their success is often influenced by various factors, including technological feasibility, market demand, and competitive conditions. This uncertainty makes it difficult for enterprises to predict the ultimate returns during the innovation process, increasing innovation risks. In such circumstances, the green credit policy plays a proactive role. This policy typically establishes specialized credit support mechanisms primarily targeting clean energy, energy conservation and emission reduction, and environmental protection technology innovation projects, providing special credit support for these projects. Through this support mechanism, enterprises can obtain more flexible and innovation-friendly financing, alleviating financial pressures during the innovation process. The support provided by the green credit policy not only offers financial support but also conveys encouragement for technological innovation at the policy level, providing enterprises with more confidence in making innovation decisions. This targeted credit support effectively mitigates the risks enterprises face during the innovation process, inspiring them to more actively engage in technological innovation. Therefore, the green credit policy not only supports the innovation of heavily polluting enterprises at the financial level, but also plays an important role in reducing the risk of innovation and promoting the application of environmentally friendly technologies, which provides strong support for the sustainable development of enterprises and the realization of social and environmental protection goals.

Heavy-polluting enterprises in China are not only vital pillars of economic development but also present significant challenges to achieve environmental protection goals. On one hand, these enterprises typically operate in energy-intensive and heavy industrial sectors, contributing significantly to China’s industrialization process and economic growth by creating a large number of job opportunities. On the other hand, as green development gradually becomes the theme of development worldwide, pollutants emitted by heavy-polluting enterprises, such as wastewater, emissions, and solid waste, not only negatively impact ecosystems but also exert greater pressure on the government in terms of environmental protection. Currently, in the new development stage, heavy-polluting enterprises in China are facing tremendous pressure for technological upgrading and industrial restructuring. The Chinese government is vigorously encouraging these enterprises to innovate technologically and adopt cleaner and more environmentally friendly production methods to meet the requirements of sustainable economic development. This implies that these enterprises need to intensify technological innovation efforts and strive for transformation to reduce their negative impact on the environment and enhance resource utilization efficiency. Technological innovation by heavy-polluting enterprises is a crucial prerequisite for achieving sustainable economic development and high-quality economic growth in China. Firstly, technological innovation helps these enterprises adopt cleaner and more efficient production processes, reducing harmful pollutant emissions and environmental pollution. Secondly, technological innovation enhances resource utilization efficiency during the production process of heavy-polluting enterprises, reducing energy and material waste. By optimizing production processes and implementing circular economy models, these enterprises reduce resource consumption while improving production efficiency, reducing dependence on natural resources, and facilitating sustainable economic development. Additionally, technological innovation facilitates the upgrading and transformation of industrial structures for heavy-polluting enterprises. By introducing environmental technologies and green industries, these enterprises can break away from traditional high-pollution and high-energy consumption production modes, transitioning to new industries based on clean production and renewable resources, thereby achieving a win-win situation for both the economy and the environment in the long term. However, as bank credit is a crucial channel for enterprise financing, heavy-polluting enterprises are typically most directly affected by green credit policies due to their higher financing needs. Therefore, in the context of the green credit policy’s increasing credit constraints on heavy polluters, whether this policy can truly play its core role, compelling these enterprises to innovate technologically and achieve transformation and upgrading, remains a topic worthy of further research and exploration.

The literature review in this study can be conducted from the following two perspectives. Firstly, as China’s financial system has progressively evolved, there has been mounting scholarly interest in both the macro and micro effects stemming from the green credit policy. At the macro level, the attention of most scholars has been drawn to investigating the impact of green credit implementation on macroeconomic dynamics and environmental safeguarding. Green credit policy, as a crucial component of the broader green financial policy landscape, can utilize credit instruments to advance energy conservation within enterprises, thereby facilitating sustainable environmental and social advancement [8]. Essentially, green credit policy embodies a mechanism for allocating credit based on ecological constraints, serving as an essential path to achieve resource-efficient allocation and alleviate the pressures of regional economic development [9]. Green credit policy, as a very important credit policy tool, can effectively protect the environment and prevent pollution, prevent national credit risks, and achieve China’s dual carbon goals [10]. At the micro level, extensive researchers have examined the effects of policy on financial institutions and companies. Some maintain that the execution of green credit policy can serve to mitigate credit risks for financial institutions, improve their reputation, and thus enhance their economic performance [11, 12]. However, an opposing viewpoint posited by other scholars asserts that the implementation of policy could potentially hamper enhancements in operational efficiency for financial institutions and even lead to a series of risks [13]. At the same time, numerous scholars have embarked on delving into the micro impact of policy on enterprises, including financing costs [14], operational performance [15], green innovation [16, 17], green transformation [18], and corporate environmental responsibility [19].

Secondly, previous literature on enterprise innovation has primarily focused on studying the factors influencing enterprise innovation. For instance, many scholars have engaged in the impact of entrepreneurial innovation spirit [20, 21], corporate culture [22], internal governance structure [23], digital financial inclusion [24], and macroeconomic environment [25] on enterprise innovation. And the majority of scholars solely assess enterprise innovation through the lens of "quantity." [2, 26, 27], with limited exploration of the inherent "quality" aspect.

In summary, certain gaps exist within the existing body of literature. First, the current research on the green credit policy in China primarily falls into two categories: macroscopic evaluations that assess the influence of the policy on environmental conservation and macroeconomics, and microscopic analyses that delve into the financial risks faced by financial institutions brought by the policy and the influence of the policy on corporate financing, emission reduction, and business performance. Few scholars have thoroughly delved into the causal identification and associated mechanisms linking green credit policy and corporate innovation. Additionally, there remains a deficiency in empirical research that investigates the microeconomic effects of policy on facilitating enterprise innovation by addressing endogeneity concerns. Second, prior studies on enterprise innovation predominantly concentrated on examining factors influencing it either from within the enterprises or the macroeconomic environment. Few scholars have explored enterprise innovation through the lens of environmental financial policies, and past researchers assessed enterprise innovation solely in terms of "quantity", without considering the dimension of "quality". Third, while "Porter’s Hypothesis" contends that appropriate environmental regulations are a driving force for enterprise innovation, prompting enterprises to participate in more innovative activities to offset the negative impacts caused by environmental regulations [28], and numerous scholars abroad have confirmed the positive effects of “Porter’s Hypothesis” on enterprise development [29–31], the research gap persists in investigating whether the policy can yield positive impacts on promoting enterprise innovation amid the backdrop of more stringent green financial policies in China. It is imperative to investigate the applicability of “Porter’s Hypothesis” within the domain of green credit in China and ascertain if China’s green credit policy effectively encourages innovation among enterprises. This exploration is critical for refining the optimization of China’s green credit policy.

To address related research gaps and enrich existing literature, this paper adopts a quasi-natural experiment approach, leveraging the " Guidelines" as the exogenous variable, to investigate the causal connection between the Green Credit Policy and the innovation of heavily polluting enterprises. The measurement of enterprise innovation in this research is approached from two distinct dimensions: "quality" and "quantity". Furthermore, this research uses two research sample data of Chinese-listed industrial enterprises from 2009 to 2019, including 2,709 companies and 2,443 companies, respectively.

This article aims to address the following issues: 1. Given the tightening of China’s Green Credit Policy, does the validity of the "Porter Hypothesis" persist? Specifically, does China’s policy retain its positive influence on the innovation of heavily polluting enterprises? 2. What is the quantifiable magnitude of the policy’s actual impact on enterprise innovation? 3. Are discernible disparities in the policy’s effects on both the quantity and quality of innovation within enterprises? If such differences persist, what factors contribute to the emergence of these disparities? 4. Are enterprises with different innovation levels consistently influenced by the policy? 5. Does the influence of the policy on enterprise innovation differ owing to heterogeneity in corporate ownership, geographic location, and size?

The importance of this study is apparent from various perspectives. Firstly, alongside China’s shift towards a "New Normal" economic phase, there is a heightened urgency for environmental preservation. This phase prioritizes quality and efficiency in economic growth, with the recognition that environmental protection is central to achieving sustainable development. In this context, the green credit policy, characterized by credit thresholds, impels heavily polluting enterprises to undergo reform and innovation. While aligned with the "Porter Hypothesis," the objectives of the green credit policy and its influence on technological innovation within heavily polluting enterprises are worth in-depth investigation. This study aims to investigate the causal connection between the green credit policy and enterprise innovation, offering a theoretical basis for refining policy implementation and presenting a viable trajectory for China to attain its sustainable development objectives. Secondly, heavily polluting companies often stand as major sources of environmental contamination, with their innovation capabilities directly impacting environmental quality enhancement. By examining the interconnection between the policy and innovation in these enterprises, we can delve into whether environmental financial mechanisms effectively steer companies toward environmentally friendly innovations. This type of research can broaden the scope of enterprise innovation, enabling heavily polluting companies to shift from their traditional role as "pollution sources" to key participants actively engaged in environmental protection.

This study stems from reflections on whether "Porter’s Hypothesis" remains applicable within the context of China’s green credit policy and existing literature gaps related to corporate innovation. Therefore, the key innovations of this research can be outlined as follows: First, this study enriches the exploration of factors affecting enterprise innovation through the lens of environmentally friendly financial policies. Specifically, this study considers the impact of green credit policy on technological innovation in heavily polluting enterprises, supplementing the existing literature on enterprise innovation with quantitative analysis. It also broadens the comprehension of the correlation between macro-environmental financial policies and micro-level enterprise conduct. Additionally, this paper extends the existing body of knowledge concerning the micro-level ramifications of the policy, with a particular focus on heavily polluting enterprises. Second, this study employs a quasi-natural experiment design, leveraging the " Guidelines" in 2012 as an exogenous variable. This methodological approach serves to mitigate potential endogeneity-related biases, thereby bolstering the robustness of the empirical results. Last, distinct from prior studies that primarily focused on quantifying enterprise innovation, this paper measures enterprise innovation from two dimensions: quality and quantity. And use two sub-samples to evaluate the impact of policy on the quantity and quality of enterprise innovation. This can more accurately evaluate the policy’s effects on both aspects of enterprise innovation, thus contributing to a more enriched research perspective regarding the influence of the green credit policy on enterprise innovation.

Following an array of regression models and robustness tests, our research findings reveal the following results: (1) The enforcement of the green credit policy significantly bolsters both the quantity and quality of innovation among heavily polluting enterprises, confirming the validity of the Porter hypothesis in China. (2) The positive effect of the policy on the quantity of enterprise innovation surpasses its favorable effect impact on the innovation’s quality. (3) Unconditional panel quantile regression results indicate that the policy exerts a significant positive effect on enterprises with lower innovation capacities, no effect on those with moderate innovation capabilities, and a significant adverse effect on those with higher innovation capacities. It demonstrates the heterogeneity of the policy’s influence on heavily polluting enterprises. (4) The policy displays a significant affirmative impact on state-owned enterprises(SOEs), large-scale corporations, and establishments positioned in China’s eastern and western regions, whereas its influence on other enterprises remains negligible.

The rest of this study is organized as follows. In Section 2, we present the institutional context and put forward our hypotheses. Section 3 outlines the empirical design and the base model construction of this paper. The primary empirical results and various robust tests are presented in Section 4. Section 5 introduces the unconditional panel quantile model, followed by the results of the heterogeneity analyses in Section 6. The discussion is for Section 7. Section 8 concludes the paper and discusses its implications.

## 2. Institutional background and hypothesis development

### 2.1 Background of the green credit policy

In July 2007, "Opinions on Implementing Environmental Policies and Regulations to Prevent Credit Risks" ("Opinions") was issued. This is the first regulatory document that specifically governs the credit operations of banks, stating that all financial institutions should strengthen credit management when reviewing applications for working capital loans from enterprises and strictly control loans for enterprises involved in environmental violations to avoid financial risks [32]. The issuance of these "Opinions" represents the initial formation of the concept of "Green Credit", even though the "Opinions" lack specific measures for putting the green credit into practice [33]. Subsequently, in 2012, China’s green credit policy underwent significant adjustments and improvements. China issued the "Guiding Opinions on Strengthening Green Credit to Support Environmental Protection Industries and Energy Conservation Projects"(“Guidelines”), which clarified the development direction and key tasks of the green credit policy. This policy document highlighted the importance of supporting projects in the environmental protection sector, increasing the allocation of green credit, and strengthening credit evaluation for environmental protection industries. Financial institutions are also required to establish special accounts for green credit and extend specialized financial support to projects conforming to green standards [34]. The release of the “Guidelines” in China in 2012 marked a milestone in the development of green credit policy. It means that China has clearly defined the concept and scope of green credit, and established a relevant policy framework [35]. Therefore, “Guidelines” is considered as an exogenous variable for the following in-depth study.

### 2.2 Hypothesis development

The Green Credit Policy remodels investment and financing mechanisms within the environmental domain. It signifies a notable expansion and innovative departure from conventional environmental regulations [26]. The policy primarily influences enterprise innovation through distinct avenues: financing, signaling, and reducing innovation risks. Firstly, financial institutions serve as one of the main sources of funding for many enterprises. The policy is an essential environmental law within the financial market. Under this policy, enterprises’ environmental performance becomes a critical criterion for credit approval by financial institutions [26]. Given the unique characteristics of heavily polluting industries in China, these larger enterprises tend to have higher gearing ratios compared to others, making them more reliant on financial institutions [36]. Consequently, in the short term, the policy leads to an immediate elevation in financing standards for these enterprises and to some extent exacerbates their difficulties in obtaining loans. To ensure their long-term development, these enterprises are compelled to engage in technological innovation and upgrade their operations to reduce pollution levels and meet the loan requirements of green credit [6]. Secondly, China currently underscores the promotion of a green economy, and the policy serves as a clear indicator of "participation in the green economy for all" [37]. This prompts consumers to supervise enterprises’ environmental practices. Under the scrutiny of the public, enterprises strive to establish a positive brand image and achieve long-term development by transforming and upgrading their existing production processes, equipment, and technologies, adopting more environmentally friendly and efficient production methods. Through technological innovation and upgrading, heavily polluting enterprises can reduce pollutant emissions, enhance resource utilization efficiency, and achieve sustainable development. Lastly, innovation is often accompanied by certain risks, requiring significant financial investment from enterprises, with uncertain outcomes [38]. This high level of uncertainty poses substantial risks to enterprise innovation. The Green Credit Policy typically establishes special credit support mechanisms to offer specialized credit assistance for projects focused on green technological innovation. These projects encompass various domains such as clean energy and energy efficiency. Through the provision of targeted credit support, the policy significantly contributes to addressing the obstacles faced by heavily polluting enterprises in the course of their innovation endeavors. Therefore, we put forward Hypotheses 1.

***Hypothesis 1***: The Green Credit Policy boosts the innovation of heavily polluting enterprises.

China is an innovative country, but still not an innovative powerhouse. The innovation dilemma faced by Chinese enterprises is mainly manifested in the low quality and insufficient success conversion rate of innovation [39]. Innovation quality refers to the overall performance of innovation in various areas such as processes, outputs, and socio-economic impact. It represents the quality of all innovation outcomes and possesses unique dynamics [40]. However, there remains a notable disparity between China’s present innovation progress and that of an innovation powerhouse. One major reason for this gap is the insufficient focus on the quality of innovation output, resulting in a situation where innovation is characterized by a high "quantity" but low "quality" [41]. Therefore, it holds vast practical importance to investigate the quantity and quality of innovation of enterprises. The Green Credit Policy offers financial backing to these firms adhering to the required standards, reducing their financing costs for innovation. This paves the way for these enterprises to amplify their investment in innovation, thus fostering an enhancement in their innovation output. [42]. In addition to financial support, the Green Credit Policy often establishes mechanisms for rewarding and evaluating green technological innovation. These mechanisms encourage enterprises to seek high-quality technical solutions and innovative achievements. Furthermore, the introduction of government monitoring and evaluation mechanisms prompts enterprises to conduct technology audits, environmental impact assessments, and other activities, further strengthening the quality of their innovation [42]. The primary mechanism through which the policy propels enterprise technological innovation is financial backing. Funds play a crucial role in stimulating enterprise innovation, which can directly affect the scale and quantity of innovation. Therefore, there is a notable surge in innovation output over the short term. However, successful innovation by enterprises requires a longer cycle and involves certain risks. Therefore, when the government actively promotes Green Credit Policy to accelerate enterprise transformation, enterprises prioritize adhering to policy and market requirements and increase the number of innovations in a short period to address the pressures imposed by the credit policy. However, this approach often lacks a long-term outlook, resulting in relatively less emphasis on the quality of their innovation endeavors. Consequently, we come up with the subsequent research hypothesis.

***Hypothesis 1a***: The Green Credit Policy can stimulate increased innovation in terms of both quantity and quality among heavily polluting enterprises, with a more substantial influence on the former.

The effectiveness of the Green Credit Policy’s positive influence may differ based on the innovation capacities of distinct enterprises. Heavy polluters with low innovation capacity typically face shortages of capital and technology and are more dependent on bank credit. Due to their lower innovation capabilities, these enterprises may have relatively weak market performance and may not possess sufficient valuable collateral or credibility to access alternative non-bank financing channels [43]. Bank credit is relatively flexible and often accepts lower collateral values, making it the primary source of financing for enterprises with lower innovation capabilities. In addition, the policy aims to help these enterprises overcome technological shortcomings by providing funding support, thereby promoting their innovation endeavors [44]. However, enterprises with high innovation capabilities may already possess great innovation capacity and market competitiveness. Consequently, they are more inclined to secure backing through alternative funding avenues, such as venture capital or direct financing [45]. In comparison, the low-cost funding provided by the policy may not be sufficiently attractive for their innovative projects and may even increase their financing costs and risks. Additionally, the policy often sets certain innovation directions for enterprises, and these constraints may impose certain limitations on their innovation orientation. Therefore, we propose Hypotheses 2.

***Hypothesis 2***: The innovation quantity and quality of enterprises with low innovation capabilities are notably positively affected by the Green Credit Policy. However, the policy shows no influence on the innovation of enterprises with high innovation capacities and could potentially lead to negative effects.

The influence of the policy on enterprise innovation can differ based on factors such as property rights, size, and geographical location. State-owned enterprises(SOEs), owing to their special political status, bear the main policy-driven economic tasks in social development. These enterprises often rely more on policy funding support and preferential credit policies [46]. Furthermore, owing to the inherent information asymmetry within the market economy, banks display a greater willingness to extend credit funds to SOEs possessing good reputations and high social trust [47]. Large-scale enterprises attract more social attention, which can reduce information asymmetry and effectively lower agency costs between the enterprise, financial market, and investors, facilitating the acquisition of various investment support [48]. Large-scale enterprises exhibit a high degree of personalized operation, and the cash flows generated from their operational activities are relatively evenly distributed. This provides an opportunity for these enterprises to create a low-cost financing structure adjustment, which contributes to their development [49]. Large-scale enterprises have accumulated extensive experience and undergone long-term growth, enabling them to rely on economies of scale to reduce production costs. They play a vital role in both job creation and economic advancement, leading financial institutions to display increased willingness to extend credit support to them. The development status of enterprise’s geographical location will influence the effect from the policy. Considerable variations in economic development are evident across various regions in China. Notably, the East enjoys the highest level of economic advancement, then the Central, with the West lagging. In economically well-developed areas, financial institutions have reached a high level of maturity, resulting in relatively lower financing costs for green credit. Enterprises can allocate funds efficiently, promoting their research and development [50]. Simultaneously, regional variations are evident in the stringency of environmental regulations. The East has always been the center of economic development with a large number of enterprises, particularly those with high pollution. Therefore, they face stricter control under the policy [51]. The West has a relatively underdeveloped financial sector, and enterprises heavily rely on bank credit for financing. Thus, the motivating impact of the policy is more pronounced for enterprises situated in the West [26]. Thus, Hypothesis 3 is put forward by us.

***Hypothesis 3***: The Green Credit Policy is more inclined to have a positive impact on innovation in large-scale enterprises, SOEs, and enterprises situated in both the East and West.

## 3. Empirical design

### 3.1 Data source

This research investigates the influence of China’s Green Credit Policy, implemented in 2012, on the sample of industrial enterprises listed in the Shanghai and Shenzhen A-share markets from 2009 to 2019 (S1 File(xls)). Rigorous measures were taken to ensure the reliability of the sample data. This involved excluding certain types of enterprises, such as ST, ST*, PT, and PT* entities, along with financial and insurance enterprises. Enterprises lacking crucial main-variable data, those with a gearing ratio exceeding 1 or falling below 0, and those displaying other abnormal financial indicators were also excluded. Ultimately, two distinct datasets were obtained. Specific data can be found in S1 File(xls). The first dataset comprised 2,709 enterprises, yielding a total of 15,805 valid observations. Meanwhile, the second dataset consisted of 2,443 enterprises, contributing to 13,030 valid observations. To mitigate the impact of extreme values, this study employed winsorization techniques on the 1% and 99% quantiles of all continuous variables. The data sources for this research primarily encompass four components. First, all enterprise-related data were derived from the CSMAR and WIND databases. Second, Regional characteristic data were acquired from the Yearbook of each province. Third, missing data for specific years were manually gathered from the RESSET database. Finally, all enterprise patent data were sourced from the CNRDS database.

### 3.2 Model construction

This paper, following the methodology employed by previous researchers [52, 53], adopts a DID(difference-in-difference) model to investigate the causal connection between the Green Credit Policy and the quantity and quality of enterprise innovation. In contrast to conventional approaches, the DID not only compares the average change in the sample before and after the policy implementation but also captures the policy’s net effect. Moreover, the DID model helps avoid issues of model endogeneity that stem from causal relationships between explanatory and dependent variables. Therefore, the DID model typically serves as a means to assess policy effects [54]. Then we establish both a treated group of industrial enterprises influenced by policy and a control group unaffected by it. The specific design of the model is detailed as follows:

$$did=Policy_{it}\;*\;Treat_{it}.$$

$$Patent_{k}=\beta_{0}+\beta_{1}\;*\;did+\theta \;*\;\sum x+\mu_{i}+\gamma_{t}+\lambda_{j}+\varepsilon_{ij}(k=1,2)$$ (1)

In this context, where, *i*, *t*, *j* respectively denote enterprise, year, and region. The *did* in this model serves as the core explanatory variable, manifested as the interaction between *policy_it_* and *Treat_it_*. *patent_k_* represents the dependent variable, with *patent*_1_ signifying enterprise innovation quantity when *k* equals 1, and *patent*_2_ denoting innovation quality when *k* is 2. The fixed effects *μ_i_, γ_t_*, and *λ_j_* correspond to individual, time, and regional factors respectively. ∑x encompasses all control variables, and the residual term is denoted by ε_*ij*_. The primary focus of this study revolves around the coefficient *β*_1_. If *β*_1_ exhibits a significant positive value, it indicates substantial promotion of both industrial enterprise innovation quantity and quality through the policy. Furthermore, to address potential issues of heteroskedasticity and serial correlation, this study employs robust standard errors with firm-level clustering for regression estimation. Subsequently, the ensuing empirical investigation delves into the examination of the policy’s influence on the quantity and quality of enterprise innovation.

### 3.3 Variables

#### 3.3.1 Explained variable

The explained variable of this research is enterprise innovation performance. This paper dissects the innovation performance of enterprises into two primary dimensions: innovation quantity and innovation quality. On the one hand, following relevant literature [55], the measurement of innovation quantity incorporates the cumulative count of successfully authorized invention patents, utility model patents, and design patents that enterprises have applied for within the current year. On the other hand, following international mainstream practices [56, 57], the assessment of innovation quality involves quantifying the number of citations or forward citations of enterprise patent data. This approach systematically scrutinizes the influence of the policy on the innovation quality of enterprises.

#### 3.3.2 Explanatory variable

In this research, we consider China’s 2012 Green Credit Policy as an external explanatory variable, which is expressed as the interaction between *Policy*_*it*_ * *Treat*_*it*_. When a given industrial enterprise is categorized as a heavy-polluting entity, *Treat*_*it*_ is assigned a value of 1. Otherwise, it takes on a value of 0. The compilation of heavy-polluting firms is founded on the revised Guidelines on Industry Classification of Listed Firms by the China Securities Regulatory Commission in 2012, along with the List of Listed Firms for Environmental Verification Industry Classification in 2008 [35, 58, 59]. This encompasses sectors such as thermal power, steel, cement, electrolytic aluminum, coal, metallurgy, building materials, mining, chemicals, petrochemicals, pharmaceuticals, light, textiles, and leather (codes: B06, B07, B08, B09, B11, C17, C18, C19, C22, C25, C26, C27, C28, C29, C30, C31, C32, C33, D44.). The variable *policy*_*it*_ signifies the time dummy variable, taking on the value 1 from 2012 onward and 0 otherwise. For simplification, we employ the term "*did*" to represent the interaction term *Policy*_*it*_ * *Treat*_*it*_, where *i* denotes different enterprises and *t* signifies time.

#### 3.3.3 Control variables

To rigorously assess the policy’s impact while minimizing the influence of external factors, the study draws on pertinent literature to employ a strategy of controlling a range of variables at both the enterprise and provincial levels [59, 60]. At the enterprise level, referring to the existing literature [14], we control the following variables: Size of the enterprise(SIZE), liabilities of the enterprise(LIABILITY), Duration of enterprise existence(AGE), The ratio of assets to liabilities(LEVERAGE), Revenue from operations (OR), Profit after tax (NP), Cash ratio of assets (CFO), Net profit as a percentage of total assets (ROA), Tobin Q(TQ), government subsidies(SUBSIDY), the largest holder rate (HOLD). At the provincial level, drawing on relevant literature [61], this paper also controls for the following variables: Degree of local economic advancement (In_PERGDP), Extent of foreign investment (FDI), Educational level within the locality (EDU), Size of the population (POP), industrial structure(IND), Consumer buying capacity (WAGE), Total value of industrial production (TOTAL). Table 1 provides the definitions of the variables.

**Table 1:** Variable definitions.

Variable classification	Variable		Variable definition
**Explained or dependent variable**	Quantity of enterprise innovation	Patent1	Total number of invention patents, utility model patents, and design patents successfully applied for and granted by the enterprise plus one(logarithmic)
	Quality of enterprise innovation	Patent2	The number of citations or forward citations of enterprise patent data plus one (logarithmic)
**Explanatory or independent variable**	Treat	Treat	If an enterprise is categorized as a heavy-polluting firm, Treat is assigned a value of 1; otherwise, it is set to 0.
	Policy	Policy	For sample years starting from 2012 and onward, the value of "post" is assigned as 1; otherwise, it is set to 0.
**Enterprise-level control variables**	Size of the enterprise	SIZE	The logarithm of the year-end total assets
	liabilities of enterprise	LIABILITY	The logarithm of year-end total liabilities
	Duration of enterprise existence	AGE	The logarithm of (Year of survey ‐ Year of establishment of the enterprise + 1)
	The ratio of assets to liabilities	LEVERAGE	Liabilities-to-Assets Ratio at the end of the year
	Revenue from operations	OR	The logarithm of the year-end operating revenue
	Profit after tax	NP	The logarithm of year-end net profit
	Cash ratio of assets	CFO	Year-end total cash as a proportion of total assets
	Net profit as a percentage of total assets	ROA	Net profit margin as a ratio of average total assets
	Tobin Q	TQ	Obtained from the CSMAR database, a metric used to assess the expansion of businesses (in logarithmic form).
	Government subsidies	SUBSIDY	Subsidies received from the government (logarithmic)
	Largest holder rate	HOLD	The ownership proportion of the major shareholder
**Provincial-level control variables**	Degree of local economic advancement	In_PERGDP	The natural logarithm of the per capita GDP in the province where the company is situated
	Extent of foreign investment	FDI	The logarithm of the total goods imported and exported by foreign-invested enterprises
	Educational level within the locality	EDU	The natural logarithm of the population with college-level education and above in the province where the enterprise is situated.
	Size of the population	POP	The logarithm of the total provincial population at the year-end.
	Industrial structure	IND	The ratio of total industrial value to the total output value.
	Consumer buying capacity	WAGE	The logarithm of the ratio between the total wages of employees in active positions and the number of employees in active positions.
	Total value of industrial production	TOTAL	The logarithm of gross industrial output.

### 3.4 Descriptive statistics

Table 2 provides an overview of the sample characteristics. The first dataset comprises a total of 15,805 samples, with 10,477 in the control group and 5,328 in the treated group. This indicates that the treated group accounts for 33.71% of the total samples. The second dataset comprises a total of 13,080 samples, including 8,383 samples in the control group and 4,697 samples in the treated group. The treated group represents 35.91% of the total samples.

**Table 2:** Main variables’ descriptive statistics.

Indus dummy	Variable	N	Nean	Standard Deviation	Minimum	Maximum
**Control group**	logpatent1	10477	2.61	1.97	0	7.12
	*did*1	10477	0	0	0	0
	Policy	10477	0.75	0.43	0	1
	Treat	10477	0	0	0	0
	logpatent2	8383	1.32	0.48	0	2.14
	*did*2	8383	0	0	0	0
	Policy	8383	0.81	0.40	0	1
	Treat	8383	0	0	0	0
**Treated group**	logpatent1	5328	2.59	1.6	0	7.12
	*did*1	5328	0.73	0.44	0	1
	Policy	5328	0.73	0.44	0	1
	Treat	5328	1	0	1	1
	logpatent2	4697	1.25	0.44	0	2.14
	*did*2	4697	0.79	0.41	0	1
	Policy	4697	0.79	0.41	0	1
	Treat	4697	1	0	1	1

Moreover, there is no significant disparity in the mean values of enterprise innovation quantity observed between the two groups. Both groups exhibit high standard deviations in innovation quantity, particularly the control group with a standard deviation of 1.97. This variability underscores the uneven nature of innovation quantity among Chinese enterprises. Regarding the quality of enterprise innovation, a noticeable difference in mean values between the two groups is observed, the mean value of innovation quality in the control group is much higher than that in the treated group. Meanwhile, the standard deviation of innovation quality in the two groups is not markedly high. Interestingly, the control group exhibits a greater standard deviation in innovation quality compared to the treated group, implying that the development of innovation quality within the control group is more uneven than that in the treated group.

## 4. Empirical results

### 4.1 Base model

The outcomes of Model 1’s regression have been presented in Table 3. Columns (1) and (2) present the regression results concerning the quantity of enterprise innovation. Column(1) depicts the regression outcomes with only independent and dependent variables. The coefficient *β*_1_ stands at 0.67 and holds statistical significance at the 1% confidence level. Transitioning to column (2), the regression takes into account control variables, clustering, Year FE, Individual FE, and Regional FE. In this case, the coefficient *β*_1_ equals 0.143 and still maintains statistical significance at the 1% confidence level. These findings underscore the significant positive influence of the Green Credit Policy on the quantity of industrial enterprise innovations. More exactly, *β*_1_ stands for when holding other variables constant, the treated group of enterprises showed an average increase of 14.3% in the quantity of innovation compared to the control group. Column(3) and column(4) present the regression outcomes concerning the quality of enterprise innovation. Column(3) is the similar to column(1). The coefficient *β*_1_ in Column (3) is estimated at 0.261, displaying statistical significance at the 1% confidence level. Column (4) aligns with column (2), revealing a coefficient *β*_1_ of 0.0289, which is statistically significant at the 5% confidence level. These results underscore the notable enhancement of the quality of industrial enterprise innovations due to the policy. 0.0289 stands for when holding other variables constant, the treated group of enterprises showed an average increase of 2.89% in the quality of innovation compared to the control group. An examination of the regression coefficients unveils that the positive impact of the policy on the quantity of enterprise innovation surpasses its positive impact on the quality of enterprise innovation. The above observations effectively validate both Hypothesis 1 and Hypothesis 1a.

**Table 3:** Base regression results.

	(1)	(2)	(3)	(4)
	logpatent1	logpatent1	logpatent2	logpatent2
***did***	0.666***	0.143***	0.261***	0.0289**
	(0.0281)	(0.0461)	(0.00929)	(0.0131)
**Constant**	2.358***	-9.153	1.155***	-1.897
	(0.0317)	(6.355)	(0.00866)	(1.889)
**Control variable**	NO	YES	NO	YES
**Cluster_Firm**	NO	YES	NO	YES
**Year FE**	NO	YES	NO	YES
**Individual FE**	NO	YES	NO	YES
**Regional FE**	NO	YES	NO	YES
**Observations**	15,805	15,560	13,080	12,821
**R-squared**	0.0041	0.857	0.007	0.851

Note: Robust standard errors in parentheses. ***, ** and * represent p<0.01, ** p<0.05, * p<0.1, respectively.

### 4.2 Robust tests

#### 4.2.1 Parallel trend test

**Fig 1:**
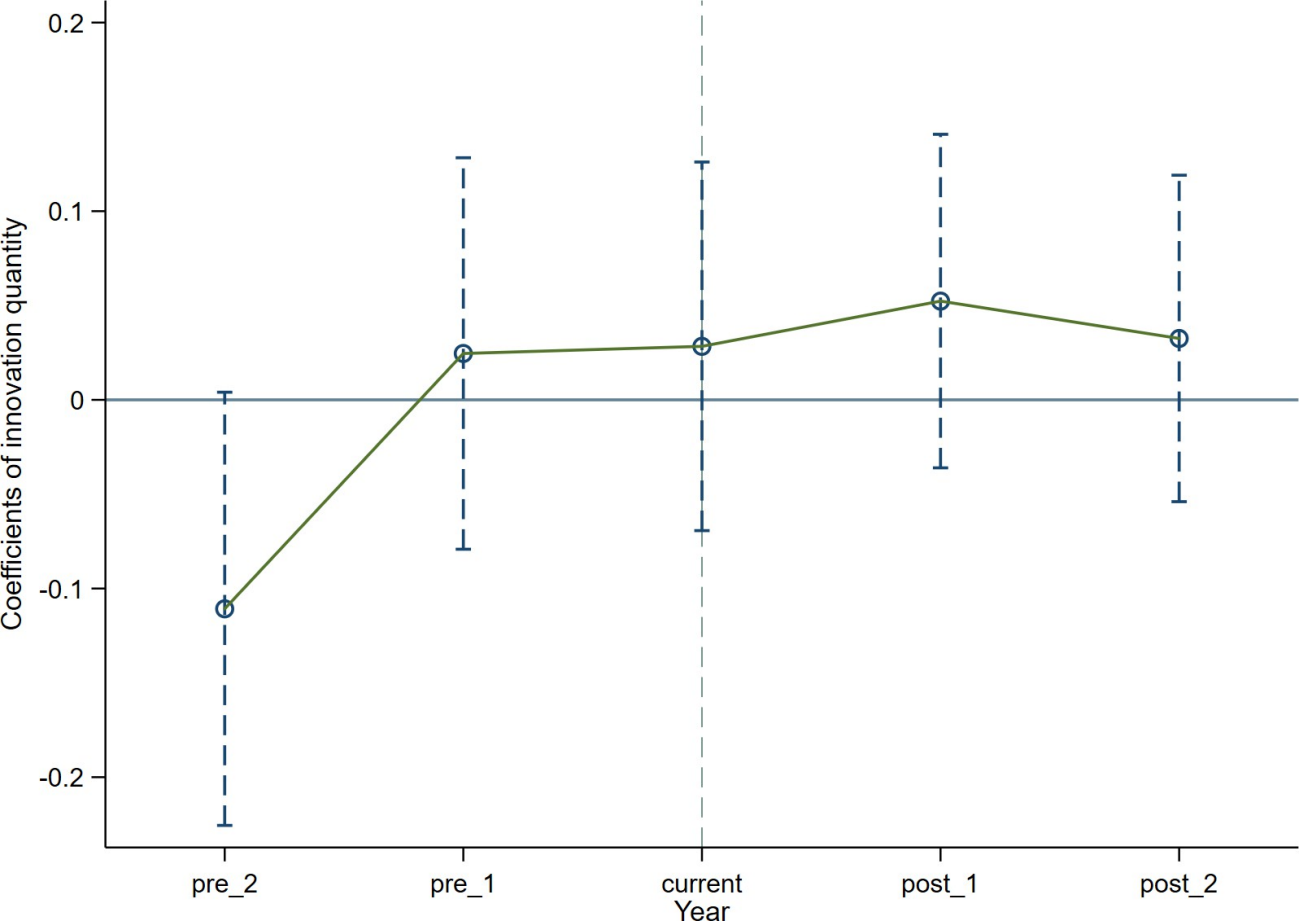
Parallel trend test (quantity of enterprise innovation).

**Fig 2:**
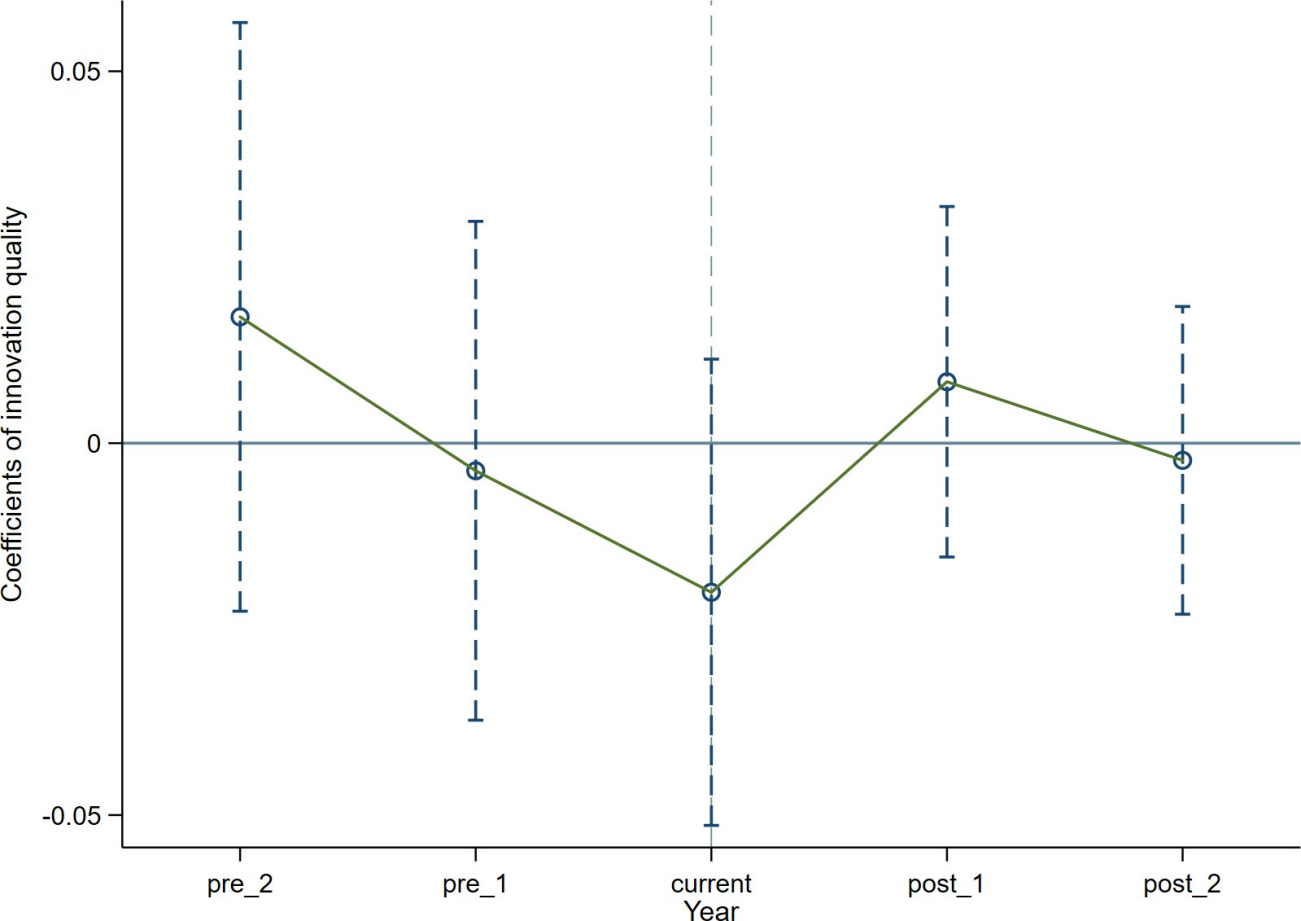
Parallel trend test (quality of enterprise innovation).

Conducting the parallel trend test is a crucial step when employing the DID method. This implies that the temporal trajectory of the control group and the treated group should display consistency prior to the policy. Consistent with previous literature [62, 63], the subsequent model is formulated. Where $did_{it}^{k}$ denotes the virtual variable. With the policy’s enactment intervention in 2012, we define *k* = *t* -2012. In addition, when conducting the parallel trend test, a common practice is to drop the base period or the previous policy treatment to avoid the influence of multicollinearity. Thus, in this model, the baseline year of policy implementation, which is 2009, is excluded. Our focus is solely on the two years preceding and the two years following the policy intervention. Coefficient *β*_*k*_ signifies alterations in enterprise innovation during this period. Coefficient *β*_0_ denotes the policy effect of 2012. The remaining variables in the model (2) are consistent with those in the base model. If coefficient *β*_*k*_ lacks statistical significance before policy intervention, it confirms that there is no substantial distinction between the two groups before policy, thus validating the assumption of parallel trends. The regression outcomes of the model (2) are exhibited in Table 4, Figs 1 and 2 respectively.


$$Patent_{k}=\alpha +\sum_{k=-2}^{k=2}\beta_{k}\;*\;did_{it}^{k}+\theta \;*\;\sum x+\mu_{i}+\gamma_{t}+\lambda_{j}+\varepsilon_{ij}\;(k=1,2)$$ (2)

As displayed in Table 4, the estimated coefficients *β*_*k*_ exhibits insignificance before policy intervention, signifying the absence of substantial differentiation between two groups before policy. Additionally, as observed from Figs 1 and 2, it is known that the 95% confidence interval of the coefficient *β*_*k*_ encompasses a value of 0, with all P-values exceeding 0.1. This reaffirms the absence of notable distinction between the two groups before 2012.

**Table 4:** Parallel trend test.

	(1)	(2)
	logpatent1	logpatent2
***pre*_2**	-0.111	0.0170
	(0.0585)	(0.0202)
**pre_1**	0.0246	-0.00371
	(0.0529)	(0.0171)
**current**	0.0284	-0.0200
	(0.0498)	(0.0160)
**post_1**	0.0524	0.00825
	(0.0451)	(0.0120)
**post_2**	0.0325	-0.00231
	(0.0441)	(0.0105)
**Constant**	-8.100	-1.806
	(6.422)	(1.889)
**Control variable**	YES	YES
**Cluster_Firm**	YES	YES
**Year FE**	YES	YES
**Individual FE**	YES	YES
**Regional FE**	YES	YES
**Observations**	15,560	12,821
**R-squared**	0.857	0.851

Note: Robust standard errors in parentheses. ***, ** and * represent p<0.01, ** p<0.05, * p<0.1, respectively.

#### 4.2.2 Adjust the temporal window

Prior to policy implementation, China recognized the importance of green finance and introduced diverse credit-related measures. Among these, the 2012 Green Credit Policy stands out as the most impactful. Therefore, to avoid the potential impact of other policies and the potential serial autocorrelation issues stemming from the extended sample duration on the empirical outcomes’ precision, referring to previous literature [60, 64], this paper presumes that the year 2010 as the implementation year for the policy. Subsequently, a counterfactual test is designed around this assumption to facilitate the regression analysis. The DID estimation results using the revised sample are presented in Column (1) and Column (2) of Table 5. These outcomes reaffirm the significant positive nature of the regression coefficient, further reinforcing the robustness of the primary regression results.

**Table 5:** Robustness examination for adjusting policy time.

	(1)	(2)
	logpatent1	logpatent2
***did***	0.173***	0.073***
	(0.0613)	(0.0223)
**Constant**	-9.766	-2.139
	(6.383)	(1.887)
**Cluster_Firm**	YES	YES
**Year FE**	YES	YES
**Individual FE**	YES	YES
**Regional FE**	YES	YES
**Observations**	15,560	12,821
**R-squared**	0.857	0.852

Note: Robust standard errors in parentheses. ***, ** and * represent p<0.01, ** p<0.05, * p<0.1, respectively.

#### 4.2.3 PSM-DID

**Fig 3:**
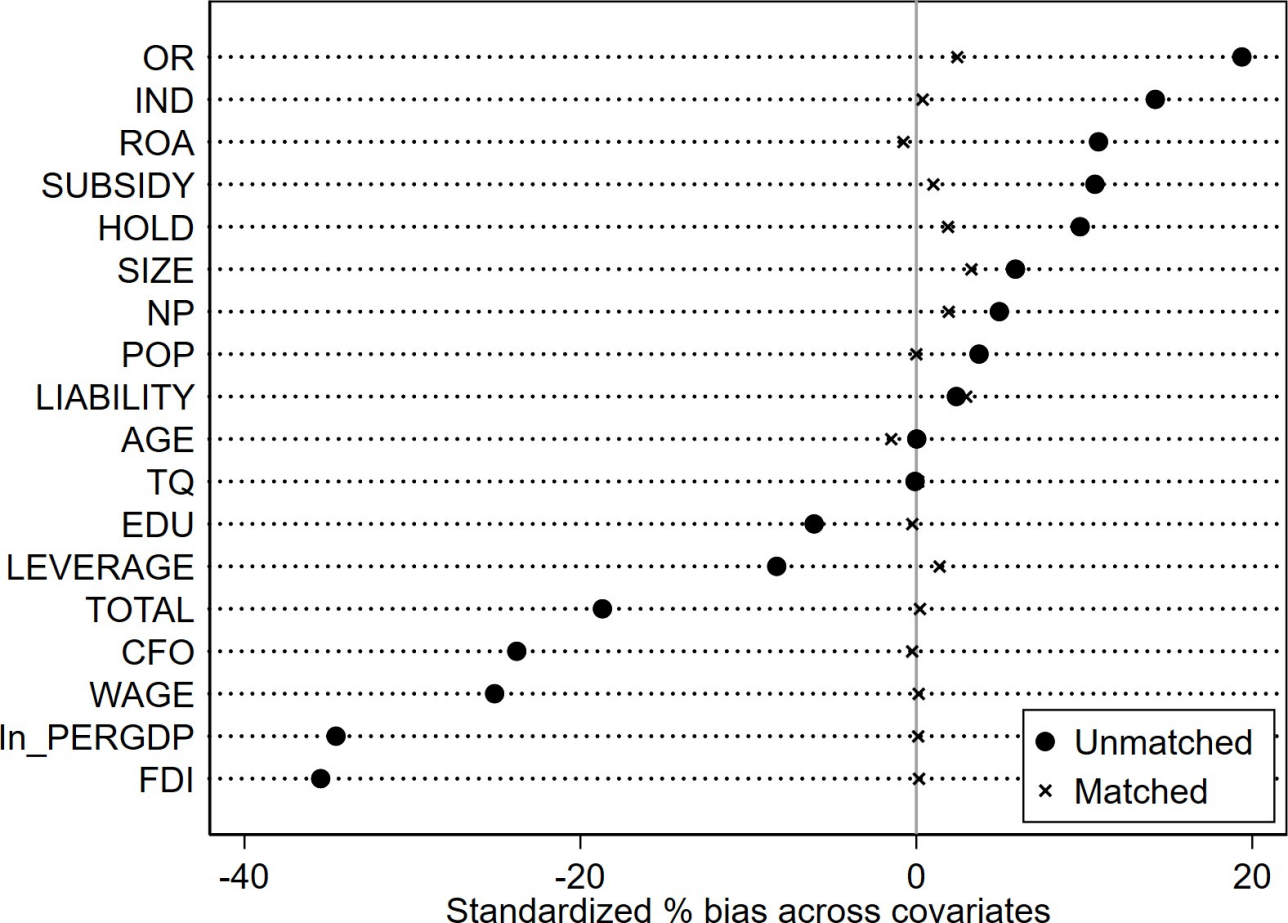
The normalized variation of the covariates (for the quantity of enterprise innovation).

**Fig 4:**
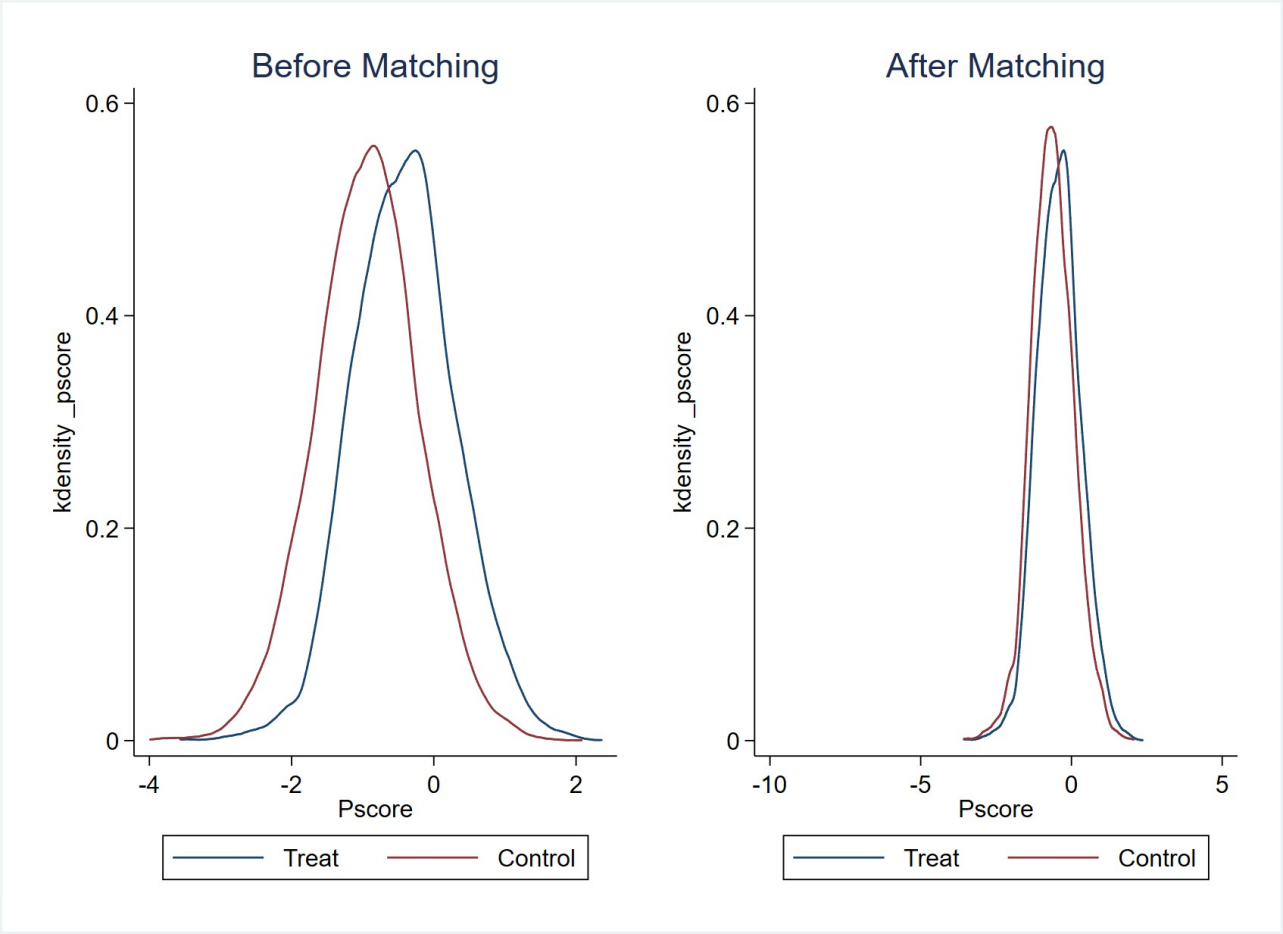
Propensity scores’ distribution (for the quantity of enterprise innovation).

**Fig 5:**
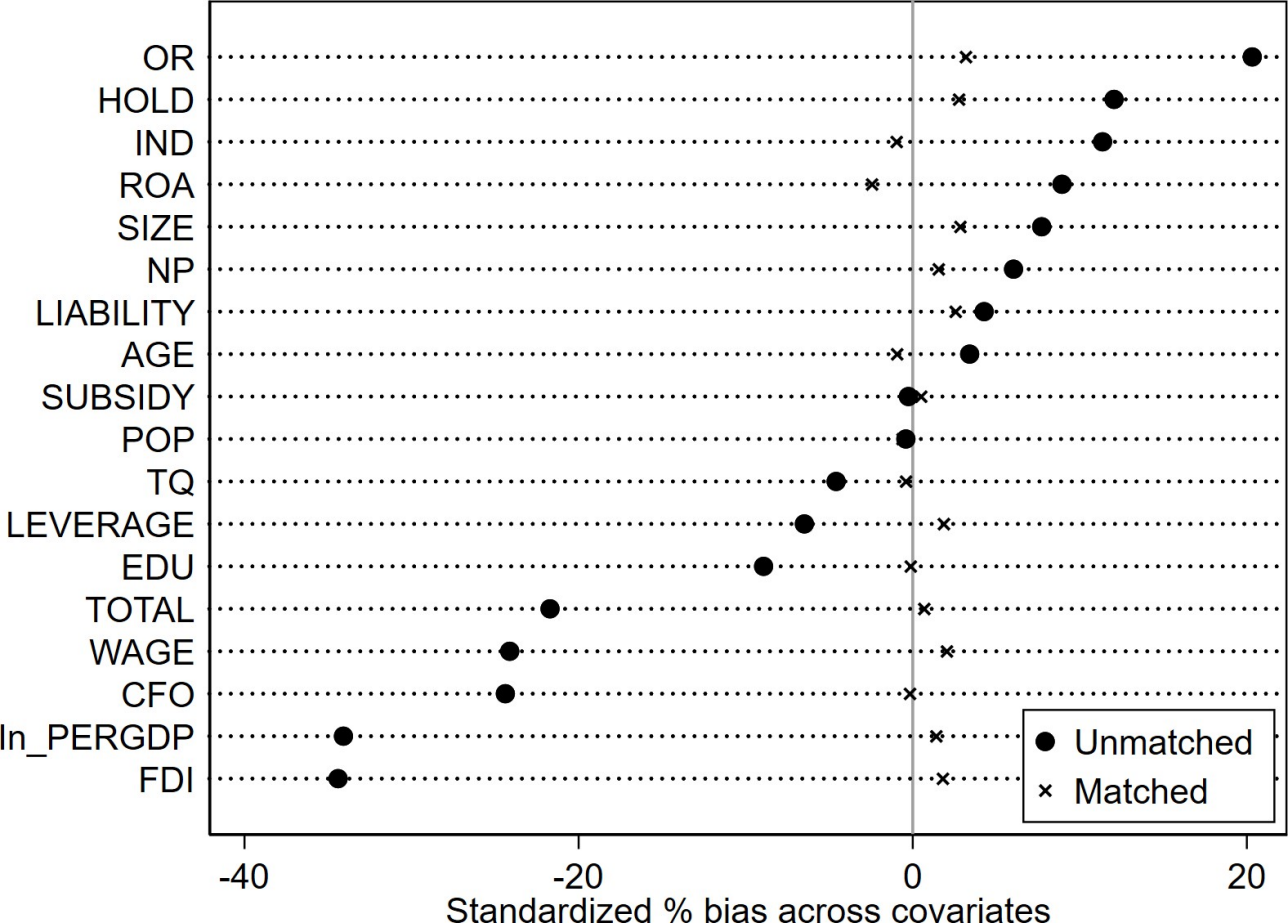
The normalized variation of the covariates (for the quality of enterprise innovation).

**Fig 6:**
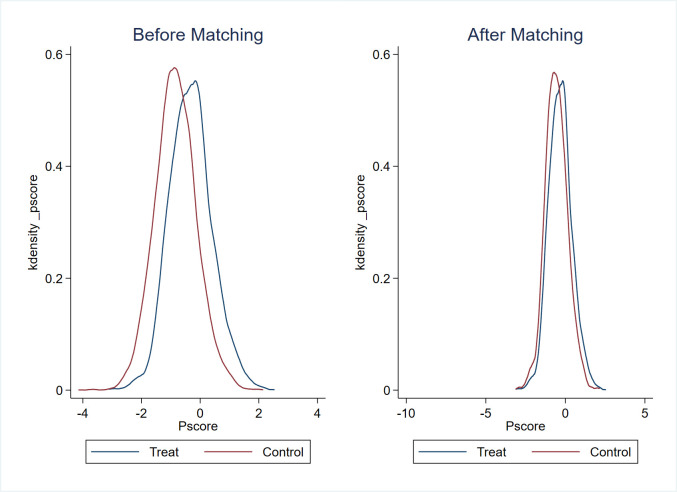
Propensity scores’ distribution (for the quality of enterprise innovation).

Given the significant diversity in terms of enterprise size, operational performance, and geographic distribution within the sample, there is a potential for the regression outcomes to be influenced by sample selection bias. Addressing this issue, we perform a robustness test using the PSM-DID approach, incorporating control variables at both the enterprise and province levels as covariates. To estimate the likelihood of each sample being assigned to the treated group, a logit model is employed. Subsequently, the nearest-neighbor (1:3) matching technique is applied to identify an appropriate control group for the treated counterparts. The effectiveness of the nearest-neighbor matching approach is vividly demonstrated in Figs 3–6, which illustrates that the standardized deviations of all variables are below 5%. The PSM-DID estimation outcomes derived from nearest-neighbor matching are outlined in Table 6, encompassing both the enterprise innovation quantity and quality, as presented in Column 1 and Column 2, respectively. In Column 1, the coefficient *β*_1_ is 0.142, exhibiting statistical significance at the 1% confidence level. Correspondingly, in Column 2, the coefficient *β*_1_ is 0.0287, holding statistical significance at the 5% confidence level. These findings further bolster the robustness of our conclusion that the implementation of the policy distinctly and positively impacts both the quantity and quality of enterprise innovation.

**Table 6:** Results from PSM-DID regression.

	(1)	(2)
	logpatent1	logpatent2
***did***	0.142***	0.0287**
	(0.0460)	(0.0131)
**Constant**	-9.018	-1.920
	(6.356)	(1.892)
**Cluster_Firm**	YES	YES
**Year FE**	YES	YES
**Individual FE**	YES	YES
**Regional FE**	YES	YES
**Observations**	15,549	12,810
**R-squared**	0.857	0.851

Note: Robust standard errors in parentheses. ***, ** and * represent p<0.01, ** p<0.05, * p<0.1, respectively.

#### 4.2.4 Placebo test

**Fig 7:**
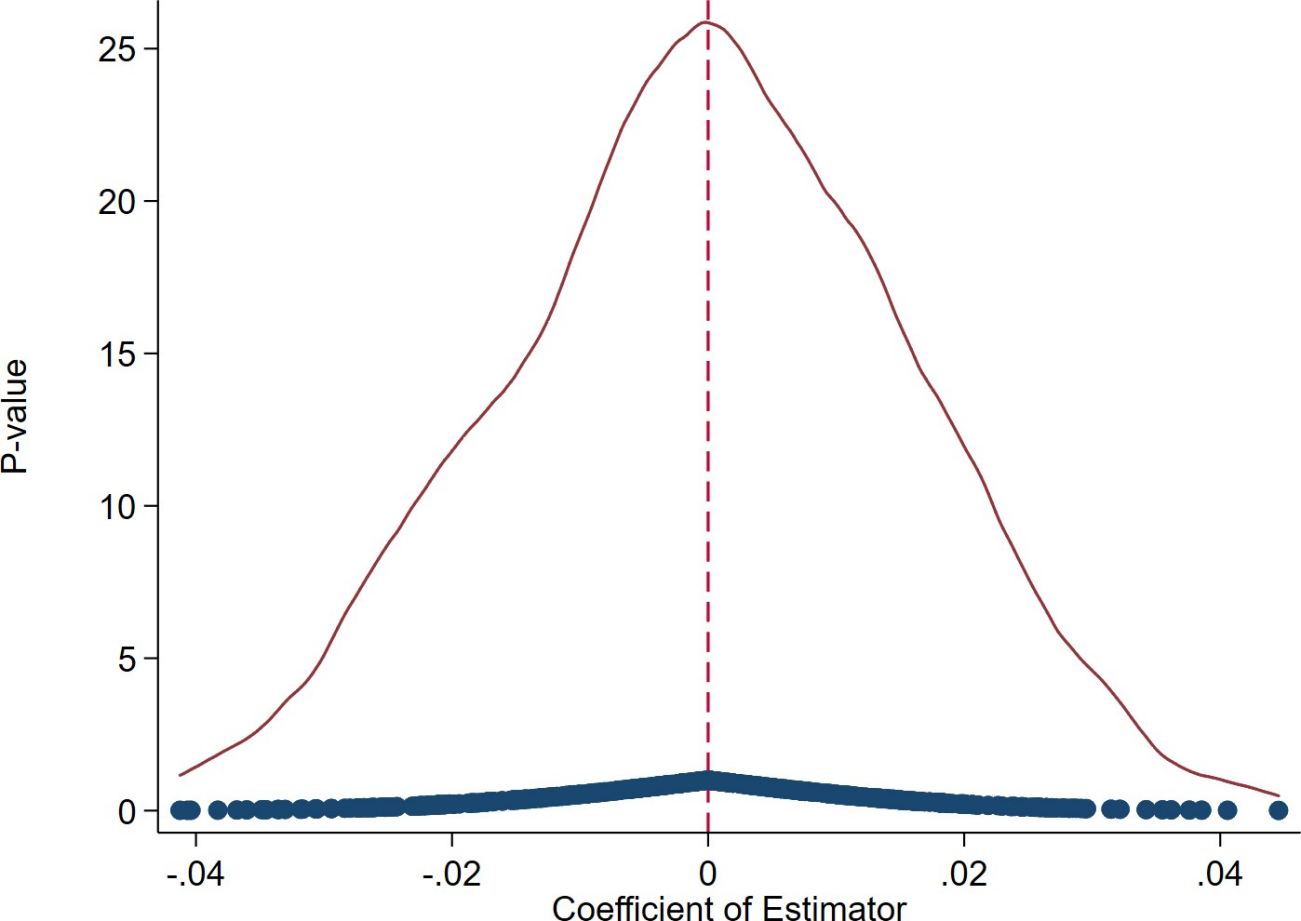
The estimated coefficients’ distribution (for the quantity of enterprise innovation).

**Fig 8:**
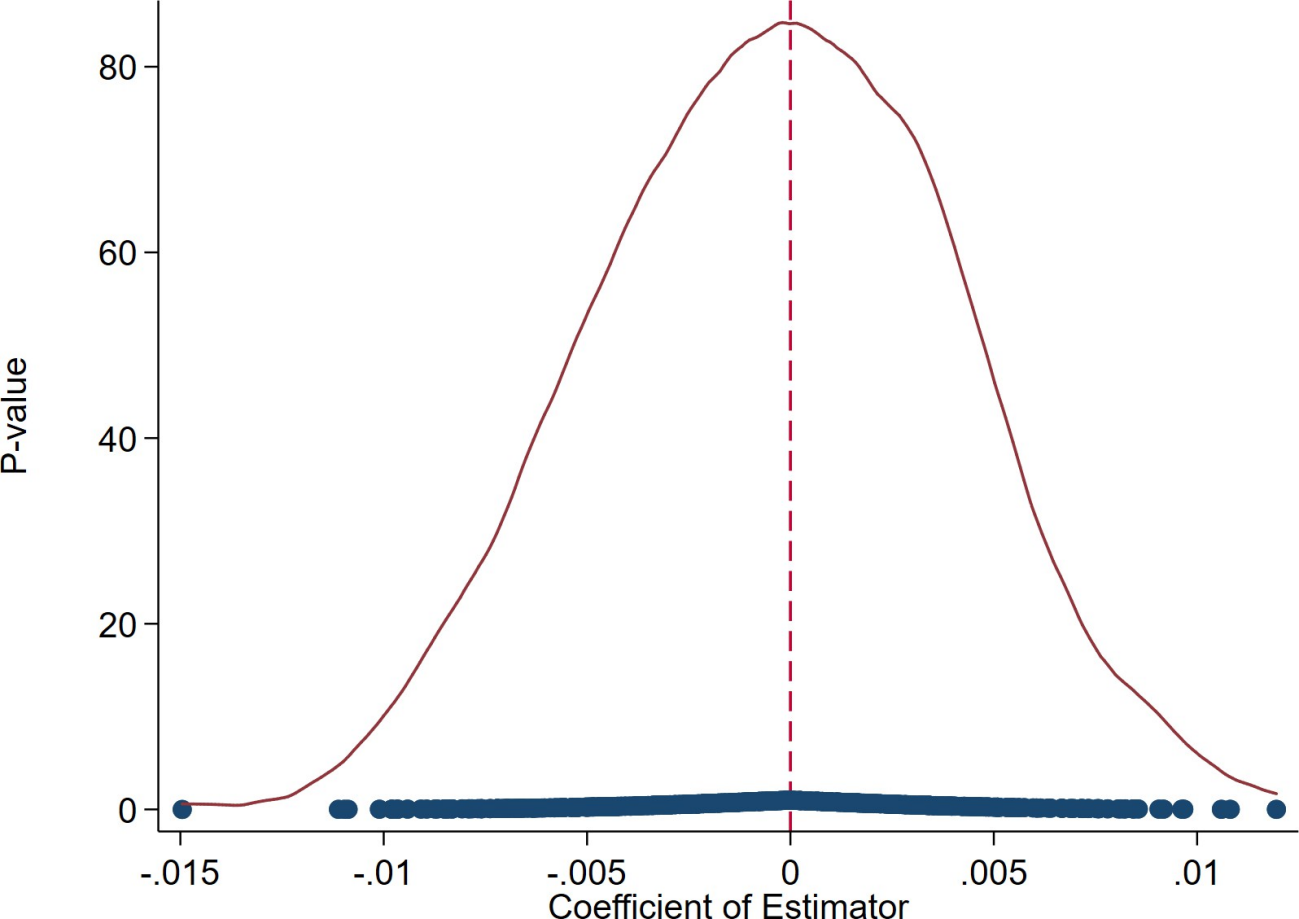
The estimated coefficients’ distribution (for the quality of enterprise innovation).

Taking into account the potential influence of random factors on accurate policy effect estimation, a placebo test is undertaken in this research using a randomization approach. Referring to the previous paper [65], a treated group for testing is formed by selecting enterprises at random. To ensure the test’s credibility, the random selection process is repeated 500 times, generating corresponding samples. Subsequently, regression analysis is conducted based on the base model. Figs 7 and 8 depict the outcomes. These graphs illustrate that the estimated coefficients of the spurious *did* from the 500 iterations tend to cluster around 0, with most coefficients failing to meet the criteria for statistical significance. Hence, we can verify that the foundational outcomes are not a consequence of random chance.

## 5. Panel quantile regression

This section employs the unconditional panel quantile regression approach to further illustrate the influence of the policy on enterprise innovation. The regression outcomes are displayed in Table 7.

**Table 7:** The unconditional panel quantile regression.

	Q25		Q50		Q75		Q90	
	logpatent1	logpatent2	logpatent1	logpatent2	logpatent1	logpatent2	logpatent1	logpatent2
***did***	0.434***	0.131***	0.413***	0.0209	0.105	-0.0427**	-0.365***	-0.115***
	(0.152)	(0.0344)	(0.0798)	(0.0191)	(0.0740)	(0.0184)	(0.107)	(0.0272)
**Cluster_Firm**	YES	YES	YES	YES	YES	YES	YES	YES
**Year FE**	YES	YES	YES	YES	YES	YES	YES	YES
**Individual FE**	YES	YES	YES	YES	YES	YES	YES	YES
**Regional FE**	YES	YES	YES	YES	YES	YES	YES	YES
**Observations**	15,560	12,821	15,560	12,821	15,560	12,821	15,560	12,821

Note: Robust standard errors in parentheses. ***, ** and * represent p<0.01, ** p<0.05, * p<0.1, respectively.

The regression results reveal that the policy’s impact on the quantity and quality of enterprise innovation is statistically significant at the 1% confidence level for the 25th quantile. On one hand, for innovation quantity, the policy maintains its significant positive impact at the 50th quantile. However, its effect becomes insignificant at the 75th quantile and shifts to a notably negative impact at the 90th quantile. Concerning innovation quality, the policy lacks a discernible effect at the 50th quantile. Interestingly, within quantiles spanning from 75 to 90, the policy manifests a substantial negative influence on innovation quality, and remarkably, the coefficient’s absolute value displays an upward trend. This trend suggests that the policy’s favorable impact on highly polluting enterprises’ innovation capacity is most pronounced among entities situated in lower quantiles. In the middle quantiles, the policy’s effect on innovation capacity is negligible, while for entities in the upper quartile, it is detrimental. In other words, the stronger an enterprise’s innovation capability, the more adverse the Green Credit Policy’s effect becomes.

Possible reasons include: First, considering the efficiency of fund allocation, enterprises with lower innovation capabilities often face a shortage of funds for innovation. For these enterprises, the low-interest and preferential loans provided by the Green Credit Policy are an important source of funding. However, enterprises with higher innovation capabilities tend to have more resources. They may prefer independent financing or alternative funding sources, reducing their reliance on the policy. Second, concerning technological thresholds and innovation capabilities, enterprises with higher innovation capacities have typically achieved some technological advancements and possess certain environmental capabilities and practices. These enterprises prioritize technological innovation rather than solely striving to meet environmental standards. Consequently, their demand for involvement in green projects supported by the Green Credit Policy might be relatively low, leading to the policy’s less significant impact. Last, from the perspective of innovation risks, innovation often accompanies certain risks. Enterprises possessing lower levels of innovation capabilities might exhibit a greater inclination to embrace the policy because it provides preferential conditions for environmental projects. On the other hand, enterprises with higher innovation capabilities may prioritize long-term competitiveness and technological advantages. When the potential benefits from environmental projects are comparatively limited, enterprises tend to distribute their resources towards domains that offer greater innovation value. Consequently, this could diminish the influence of the policy on their innovation capacities.

## 6. Heterogeneity analysis

### 6.1 Analysis of enterprise-ownership heterogeneity

The preceding sections have demonstrated the substantial positive influence of the policy on enterprise innovation. Nonetheless, the outcomes of policy implementation could exhibit heterogeneity based on distinct enterprise characteristics. Hence, this study differentiates between enterprises based on their ownership attributes to conduct a more in-depth investigation into the effects of the policy.

Given the considerable variations concerning resource accessibility and policy inclinations between SOEs and Non-SOEs, this study undertakes the task of segregating the sample data into two distinct subsets: SOEs and Non-SOEs. The regression outcomes of this division are in Table 8. Within Table 8, Columns (1) and (2) display the results for the quantity of enterprise innovation, and Columns (3) and (4) present the regression outcomes regarding the quality of enterprise innovation. The findings reveal that within the subset comprising SOEs, the regression coefficient for the magnitude of enterprise innovation stands at 0.24, and its statistical significance is at the 1% level. Simultaneously, the regression coefficient corresponding to the quality of enterprise innovation is measured at 0.0464, demonstrating statistical significance at the 5% level. Conversely, for the subset encompassing Non-SOEs, none of the regression coefficients attain statistical significance. These results demonstrate the substantial positive influence of the policy on both the quantity and quality of innovation within SOEs, whereas the policy shows no discernible impact on Non-SOEs. Moreover, interestingly, the coefficient reflecting the policy’s effect on the innovation quantity of SOEs is notably greater than the coefficient for its impact on innovation quality. This implies a relatively limited efficacy of the policy in enhancing the innovation quality of SOEs.

**Table 8:** Analysis of enterprise ownership heterogeneity.

	SOE	Non-SOE	SOE	Non-SOE
	logpatent1	logpatent1	logpatent2	logpatent2
***did***	0.242***	0.0227	0.0464**	0.00942
	(0.0658)	(0.0612)	(0.0190)	(0.0185)
**Constant**	-3.566	-21.70**	-2.546	-1.779
	(7.871)	(10.68)	(2.395)	(3.251)
**Cluster_Firm**	YES	YES	YES	YES
**Year FE**	YES	YES	YES	YES
**Individual FE**	YES	YES	YES	YES
**Regional FE**	YES	YES	YES	YES
**Observations**	6,874	8,607	5,353	7,390
**R-squared**	0.884	0.835	0.869	0.836

Note: Robust standard errors in parentheses. ***, ** and * represent p<0.01, ** p<0.05, * p<0.1, respectively.

Possible explanations are as follows: First, SOEs have an important position in China’s national economy, and the government usually gives more policy support and resource priority to them. During the execution of the policy, SOEs are inclined to receive enhanced policy backing from the government, which encompasses increased green credit availability and additional supportive measures [66]. Second, the Green Credit Policy could potentially give preference to SOEs in aspects concerning the allocation of resources and entry prerequisites. SOEs usually possess greater financial and overall resources, thereby enabling them to more readily fulfill the environmental project standards imposed by the policy [67]. In contrast, Non-SOEs may face difficulties such as funding shortages and higher technological thresholds, limiting their innovation capabilities. Third, SOEs bear more social responsibilities and consider long-term development in the Chinese economy [68]. The environmental projects advocated by the Green Credit Policy often require longer return cycles and higher environmental risk-taking capacity. Owing to their strong governmental affiliations and strategic orientation, SOEs are inclined to take on such risks. Last, the enhancement of innovation quality requires more market feedback and technological optimization, accompanied by greater uncertainty and risks. Consequently, driven by the impetus of the policy, SOEs are inclined towards prioritizing the augmentation of green innovation project numbers to meet policy objectives [69].

### 6.2 Analysis of enterprise-scale heterogeneity

Differences in resources, capabilities, and innovation atmosphere among enterprises of different scales may lead to varying effects of the policy. Large-scale enterprises possess greater financial, technological, and human resources, enabling them to allocate more resources to innovation endeavors and exhibit enhanced research and development capabilities. Conversely, small-scale enterprises commonly face resource and capacity limitations, rendering them unable to undertake extensive innovation initiatives on the same scale [70]. Furthermore, in terms of organizational structure, large enterprises generally have more sophisticated systems, allowing for better organization and coordination of innovation activities. They are better equipped to translate innovation into commercial value. In contrast, small enterprises, due to their smaller size, often grapple with organizational coordination and face challenges in innovation management [71]. Lastly, in terms of innovation risk-taking, large enterprises typically possess stronger financial strength and market position, allowing them to bear the risks associated with innovation [72].

In light of this context, the present study classifies the sample data into two distinct subsets: enterprises of large scale(those with asset sizes surpassing the median total assets of the sample companies) and enterprises of small scale (those with asset sizes below the median total assets of the sample companies). Table 9 displays the outcomes for enterprise innovation quantity in Columns (1) and (2), while Columns (3) and (4) exhibit the regression results concerning enterprise innovation quality. The outcomes underscore that the policy exerts a significant and affirmative influence on the innovation quantity and quality of large-scale enterprises, with coefficients amounting to 0.382 and 0.0823, respectively, both of which maintain statistical significance at the 1% level. In contrast, the policy’s impact on the innovation quantity and quality of small-scale enterprises is not statistically significant. It is also of note that the coefficient reflecting the effect of the policy on the innovation quantity of larger-scale enterprises is notably greater than that observed for innovation quality.

**Table 9:** Analysis of enterprise-scale heterogeneity.

	Large	Small	Large	Small
	logpatent1	logpatent1	logpatent2	logpatent2
***did***	0.382***	0.00385	0.0823***	0.00871
	(0.0760)	(0.0522)	(0.0198)	(0.0179)
**Constant**	-1.772	-17.33**	-1.258	-3.600
	(8.389)	(8.214)	(2.556)	(3.190)
**Cluster_Firm**	YES	YES	YES	YES
**Year FE**	YES	YES	YES	YES
**Individual FE**	YES	YES	YES	YES
**Regional FE**	YES	YES	YES	YES
**Observations**	7,665	7,550	6,316	6,206
**R-squared**	0.892	0.822	0.888	0.818

Note: Robust standard errors in parentheses. ***, ** and * represent p<0.01, ** p<0.05, * p<0.1, respectively.

The potential explanation lies in the resource advantages and technological capabilities possessed by large-scale enterprises. These factors enable them to more readily fulfill the lending prerequisites set by the policy. Consequently, such enterprises possess more financial resources conducive to innovation endeavors [73]. However, enhancing the quality of innovation requires more advanced technologies and long-term investment in R&D, which also comes with higher risks. This poses challenges for large-scale enterprises. Consequently, when choosing innovation activities, they may prefer options that require less investment and carry lower risks, focusing on increasing innovation quantity first to enhance overall competitiveness [69].

### 6.3 Analysis of regional heterogeneity

The degree of enterprise innovation might be influenced by the level of economic development in the location where the enterprise is situated. Thus, to obtain more precise policy effects, investigating the regional variations in policy effects becomes imperative. This study categorizes enterprises into three distinct subgroups: Eastern, Central, and Western regions. This categorization is based on the varying economic development levels of their respective locations [59]. The Eastern region encompasses areas such as Beijing, Tianjin, Shanghai, Hebei, Shandong, Jiangsu, Zhejiang, Fujian, Guangdong, Hainan, Heilongjiang, Jilin, and Liaoning. The Central region includes provinces like Shanxi, Anhui, Henan, Hubei, Hunan, and Jiangxi. Lastly, the Western region includes regions such as Inner Mongolia, Chongqing, Sichuan, Yunnan, Guizhou, Tibet, Shaanxi, Guangxi, Gansu, Qinghai, Ningxia, and Xinjiang. The outcomes, as illustrated in Table 10, provide insights into the effects of the policy. Columns (1), (2), and (3) in Table 10 detail the results concerning the quantity of enterprise innovation. Columns (4), (5), and (6) present the regression outcomes for the innovation quality. The regression results indicate that the Green Credit Policy significantly fosters increased innovation quantity for both Eastern and Western enterprises. However, this effect is not statistically significant for Central region enterprises. Regarding innovation quality, the policy leads to significant improvements in the East but does not exhibit the same impact on innovation quality for enterprises in the West and Central.

**Table 10:** Analysis of regional heterogeneity.

	East	Central	West	East	Central	West
	logpatent1	logpatent1	logpatent1	logpatent2	logpatent2	logpatent2
***did***	0.101*	0.188	0.202*	0.0428***	0.00448	-0.0172
	(0.0531)	(0.117)	(0.119)	(0.0159)	(0.0325)	(0.0328)
**Constant**	-1.067	14.96	-12.00	-3.085	-16.61	-0.0186
	(9.894)	(30.16)	(18.44)	(2.738)	(10.31)	(6.142)
**Cluster_Firm**	YES	YES	YES	YES	YES	YES
**Year FE**	YES	YES	YES	YES	YES	YES
**Individual FE**	YES	YES	YES	YES	YES	YES
**Regional FE**	YES	YES	YES	YES	YES	YES
**Observations**	10,919	2,404	2,229	9,078	1,995	1,741
**R-squared**	0.864	0.850	0.841	0.854	0.851	0.847

Note: Robust standard errors in parentheses. ***, ** and * represent p<0.01, ** p<0.05, * p<0.1, respectively.

Possible reasons for this are as follows: Generally, the East exhibits a higher level of economic advancement and possesses a more substantial reservoir of innovation resources and infrastructure. In contrast, the Western region has historically experienced relatively less economic development. Nevertheless, nowadays, China has undertaken proactive initiatives to foster the progress of the West, encompassing the substantial development of green industries [74]. Simultaneously, it is worth noting that due to limited financial resources, enterprises situated in the West may lean more heavily on bank credit [75]. Consequently, the policy exerts a comparatively more significant influence on enterprises located in both the East and West.

## 7. Discussion

After conducting main regression and many robustness tests, the affirmative impact of the Green Credit Policy on both the quantity and quality of enterprise innovation has been validated using the DID model. Additionally, this research observes that the policy’s favorable impact on the quantity of enterprise innovation outweighs its impact on the quality of such innovation. Moreover, employing unconditional panel quantile regression, this investigation reveals that the policy’s positive effect on heavily polluting enterprises with distinct innovation capabilities exhibits heterogeneity. The comparisons between this paper and previous literature are as follows:

First, previous studies only use the "quantity" of innovation as a measure of enterprise innovation to study factors affecting enterprise innovation, neglecting the diversity and complexity of innovation and failing to provide a comprehensive assessment of enterprise innovation [76, 77]. This paper undertakes a thorough analysis of the impact of the policy on enterprise innovation, considering both the aspects of "quantity" and "quality". By merging investigations into these dual dimensions, a more comprehensive assessment of the policy’s impact on enterprise innovation is attained, which enriches the research perspective of promoting enterprise innovation through the Green Credit Policy.

Second, it is observed that enterprises with lower innovation capacities experienced a significant positive impact from the policy. However, enterprises with medium innovation capabilities do not show any significant effect from the policy, and enterprises with higher innovation capabilities even experience a significant negative effect. Previous studies have often studied enterprise innovation capability as a whole, neglecting the heterogeneity of enterprise innovation capabilities [78, 79]. This limitation obstructs the identification of potential differences in the influence of independent variables on dependent variables across diverse conditions and renders estimation outcomes sensitive to sample outliers. To comprehensively comprehend the policy’s influence on enterprises with varying innovation capacity levels and better capture data distribution’s asymmetry and tail effects, this investigation adopts the unconditional panel quantile regression methodology. By evaluating regression coefficients across distinct quantiles, it unveils whether the policy’s effects diverge for enterprises with low, moderate, and high innovation capabilities, thereby deepening insights into the policy’s impact and mechanisms. Furthermore, by estimating regression coefficients across different quantiles, this research can assess whether the policy yields divergent effects on enterprise innovation quantity and quality, thereby unveiling the policy’s asymmetric impact on these facets. These efforts contribute to an enhanced insight into the role of the Green Credit Policy in fostering enterprise innovation, offering valuable guidance for the formulation and implementation of policies in practical contexts.

Notably, the findings of this paper reveal that the policy has a significantly detrimental impact on enterprises possessing high innovation capabilities. This finding contradicts the prevailing views in the previous literature, which generally suggests that the policy can promote all enterprises’ innovation activities [80–82]. This finding serves as a reminder to policymakers that when formulating green credit policies, they need to consider the differences in innovation capabilities among different enterprises. This is necessary to avoid imposing unnecessary restrictions on enterprises with higher innovation capabilities and to promote innovation and transformation across all enterprises.

## 8. Conclusions and implications

This study utilizes the "Green Credit Guidelines" in 2012 to set up a quasi-natural experiment. Drawing from two distinct databases, encompassing data on the innovation quantity of 2,709 listed companies in Shanghai and Shenzhen A-shares in China spanning 2009 to 2019, as well as data on the innovation quality of 2,443 listed companies in the same regions and timeframe, this research empirically investigates the impacts of the policy on both the quantity and quality of enterprise innovation. The findings indicate a significant enhancement in the innovation capabilities of China’s heavily polluting enterprises as a result of the implementation of the " Guidelines". In addition, the policy’s positive influence on the quantity of enterprise innovation surpasses its effect on innovation quality. Furthermore, the " Guidelines " have differential effects on enterprises based on their levels of innovation capabilities. Enterprises with lower innovation capacities experienced a significant positive impact from the policy. However, enterprises with medium innovation capabilities do not show any significant effect from the policy, and enterprises with higher innovation capabilities even experience a significant negative effect. Ultimately, this investigation unveils that the policy’s affirmative influence is more significant on larger enterprises, SOEs, and companies situated in the West and East. Conversely, there is no impact observed for other enterprises due to the policy.

The conclusions drawn from this study hold substantial significance for both researchers and managers. In theoretical terms, this study confirms the proposition that China’s green credit policy effectively boosts enterprise innovation and validates the applicability of the "Porter Hypothesis" within the framework of China’s stringent green credit regulations, which offers certain academic support for future researchers in exploring Porter’s Hypothesis in the Chinese economic environment. In addition, this study analyzes the causal relationship between China’s environmental finance policy and corporate innovation from different innovation dimensions (quantity and quality of corporate innovation). It not only enriches the assessment methods of corporate innovation but also helps to better grasp the diversity and complexity of corporate innovation processes, providing future researchers with new research perspectives and evaluation methods of enterprise innovation. Finally, this research serves to enhance and broaden the existing body of knowledge concerning the microeconomic implications of the green credit policy and enterprise innovation. This study supplements the current research focused on the microeconomic consequences of the green credit policy within individual enterprises. It also enriches the body of knowledge regarding factors that influence enterprise innovation, providing a new macro-level insight for future scholars to study enterprise innovation behavior.

In terms of practice, this paper also holds some practical significance. The environmental pollution problem caused by over-industrialization has always been the key to impeding the sustainable progress of developing nations and remains the fundamental source of their environmental challenges. At present, China is undergoing a critical phase of transition from rapid growth to high-quality development in the economy. Investigating the causal link between the green credit policy and innovation within heavily polluting enterprises can point out a new achievable path for achieving sustainable development. Furthermore, it can offer specific reference value to other developing nations aiming to attain environmentally sustainable growth.

Therefore, this paper proposes some recommendations: First, from the government’s perspective, considering the favorable impact of the "Green Credit Guidelines", the government should persist in refining and implementing the policy to boost innovative endeavors among enterprises. To improve innovation quality, the government can encourage enterprises to prioritize technological innovation by strengthening policy guidance, providing technical advice, and providing training support. During this progression, it is imperative for the government to fully account for the diversity inherent in enterprise innovation and formulate distinct policies based on the innovative capabilities of enterprises. For enterprises with low innovation capacity, the government can urge financial institutions to provide more flexible and convenient credit support and appropriately lower the credit threshold to help enterprises obtain the funds they need. For enterprises with strong innovative capacity, policies should focus more on incentivizing innovation and providing the necessary technical support, while reducing unnecessary credit constraints. In addition, when implementing the policy, the government must consider variations in the geographical locations of enterprises, the ownership structures, and the sizes of the enterprises. The government could offer increased financial and technical support to smaller Non-SOEs situated in economically disadvantaged regions. For other enterprises, policy flexibility and differentiation should be enhanced. Second, from enterprise managers’ perspective, enterprises should consider the sustainable development of enterprises and their social responsibility from a long-term perspective when formulating strategies and actively supporting the national policy of environmental protection. In light of the findings from this research, corporate managers should take an active role in endorsing and engaging with green credit initiatives. They should take proactive measures to conserve energy, lower emissions, and enhance the enterprise’s technological innovation capabilities, thereby making them eligible for credit support from financial institutions. Given that the policy’s effect on enterprises may vary based on their distinct innovation capabilities, targeted recommendations can help enterprise managers better utilize policy support to promote innovation. For enterprises with low innovation capacity, they can strengthen technical training and support, and seek collaborative partnerships to enhance their innovation capabilities. For enterprises with medium and higher innovation capacity, emphasis is advised on innovation quality, risk assessment, and diverse financing. Given the heterogeneous influence of policy based on geographical location, ownership attributes, and scale, managers should comprehend the policy implications within their respective regions, optimize financing structures, strengthen innovation management, and continually focus on policy dynamics.

There are some inherent limitations to this study. First, owing to data availability constraints, the sample exclusively encompasses listed heavy-polluting enterprises, excluding their unlisted counterparts. Incorporating data from non-listed heavy-polluting industrial enterprises in the future would considerably enhance result reliability. Second, the dataset solely originates from the Chinese A-share market, thereby necessitating further validation of the findings’ applicability to diverse countries and regions. Subsequent research could broaden the sample scope to encompass other countries and regions, facilitating a more comprehensive assessment of policy implementation effects. Last, as this study focuses solely on the largest component of green finance, namely green credit, to explore its microeconomic impacts, future investigations could comprehensively evaluate green finance by assigning weightage to other green financial products like green bonds and green insurance. This approach would provide a more profound analysis of the microeconomic effects of policy interventions.

## Supporting information

S1 File (XLSX)
